# Intergenerational Synchrony and Its Effect on Bonding and Group Closeness among Young and Older Adults

**DOI:** 10.3390/bs14070607

**Published:** 2024-07-17

**Authors:** Assaf Suberry, Ehud Bodner

**Affiliations:** 1 Department of Social & Health Sciences, Bar-Ilan University, Ramat Gan 5290002, Israel; ehud.bodner@biu.ac.il; 2 Music Department, Bar-Ilan University, Ramat Gan 5290002, Israel

**Keywords:** dance, intergenerational interventions, openness to experience, social bonding, synchrony, aging, group processes

## Abstract

To examine the effect of synchronous dance movements on social bonding and perceived closeness between generations, 168 young (20–45 years) and older (65–90 years) participants were randomly assigned to six dyad conditions. These included dancing synchronously or asynchronously with an in-age-group or out-age-group unfamiliar partner for 11 min. The participants then completed social bonding and group closeness questionnaires. To assess variation across individuals’ and dyads’ measurements, a generalized estimating equation modeling analysis was conducted. In line with the hypotheses, synchronized dancing increased social bonding, and young adults showed an enhanced perception of closeness between generations. The hypothesis that synchronous dancing with out-age-group members would foster greater perceived closeness compared to in-age-group members was not confirmed. Surprisingly, the results indicated that asynchronous movements with the in-age-group led to a higher degree of closeness between generations than asynchronous movements with the out-age-group. Avenues for future studies on the mechanisms by which intergenerational dance fosters intergenerational bonding and closeness are discussed.

## 1. Introduction

The steadily growing proportion of people over the age of 60, from 12% to 22% by 2050 [1], makes ageism a problematic social issue [2]. In Israel, the older adult population (over 65) is expected to reach 14% by 2040 [3]. Older people are often stereotyped as incompetent, boring, slow, and fragile [4]. Age segregation in many life domains (i.e., education, family, employment, and retirement) between younger and older generations is considered a prominent cause for such views [5].

In response to the devastating outcomes of age segregation, intergenerational encounters for bridging the gap between generations are constantly being constructed [6]. While these encounters demonstrate effectiveness, they seem to be more efficient among adolescents and young adults than older adults [7]. Moreover, not all interventions correspond to the requirements that the interventions be individualized, based on equal-status relations and in-person intergenerational contact [6], and include shared tasks [8,9]. In the present study, we implemented these requirements and aimed to assess whether the shared performance of synchronized dance movements in dyads improves social bonding (interpersonal level) and intergenerational closeness (social level).

Two key theories (which were not examined in this study) explain how synchronous movement can foster closeness: (1) Tarr et al. [10] suggest that moving in synchrony may trigger the release of endorphins, which trigger positive feelings and a sense of reward, thereby strengthening the social bond between participants; (2) other researchers [11] suggest a perception–action mechanism behind the synchronized movements, enhancing one’s memory [12], attention [13,14], and ability to predict the movements of the synchronized person [15]. This leads to a mutual sense of trust, increased similarity, and even blurred self–other boundaries, which increase the sense of closeness between synchronized partners [16,17,18]. 

This line of research on the perception–action mechanism is based on a socio-cognitive approach showing that mimicry during dyadic interactions facilitates assessments of social connectedness and rapport [19]. It was found that people tend to unconsciously synchronize with the postures, facial expressions, and gestures of their partners and even mimic their speech accents (for a review, see [20]). This natural tendency to perform similar gestures and movements to the other person, which is also known as the “chameleon effect”, drives people to become socially closer and increases interpersonal liking and group affiliation [21]. As for the role of musical elements in the creation of coordinated movements, rhythm was found to play a major role in facilitating coordinated movements (for an extensive review, see [22]). Moving according to the same rhythm (e.g., the same beat), generated by either metronomes or musical stimuli, enables stable and predictable motor patterns during a social interaction [23]. In a study where the hand movements of the participants and the experimenter were synchronized, the memory for the experimenter’s utterances and appearance was enhanced (compared to an unsynchronized condition) [24]. Similar evidence was found in group dance settings [12]. Thus, synchronized movements appear to strengthen the fundamental aspects of interpersonal interaction, potentially influencing how we perceive not only specific others, but also individuals from different age groups.

What are the social benefits of synchronous human movements? Interpersonal synchrony is defined as “coordinated temporal movements between two or more individuals” [25] (p. 123). To test the social effects of interpersonal synchrony, researchers have manipulated auditory stimuli while comparing synchronous and asynchronous conditions. A common paradigm for the creation of asynchronous dancing movements is the silent disco paradigm [26]. Researchers who implemented this paradigm found that a condition of synchronized full-body dance movements (same music and same movements) increased social bonding between participants, compared to a condition of partially synchronized dance movements (same music, different movements) [10]. In the current study, this paradigm was implemented, and dancing movements were modified to a sitting position to ensure the safety of the older participants. 

The application of synchronous manipulation, however, extends beyond dancing movements. Prior research demonstrated its effectiveness in fostering social bonding during mere walking and finger tapping. The participants who were instucted to walk in synchrony felt more connected and trusted each other more than those who walked asynchronously [27]. Tapping in synchrony with others resulted in higher ratings of affiliation with the experimenter compared to asynchronous tapping [28]. Moreover, synchronized series of movements fostered various prosocial attitudes (such as closeness, perceived similarity, liking, affiliation, and trust), encouraged prosocial behaviors such as cooperation [29], and increased social bonding with ingroup [30] and outgroup members [31,32,33]. Additionally, walking in synchrony with an outgroup member decreased prejudice towards that outgroup [34]. Finally, qualitative research [35] suggested that dance between adult granddaughters and their grandmothers could contribute to stronger intergenerational bonds. Therefore, building on the established literature regarding the synchrony-bonding effect [18,27,28,29,30,31,32,33,34,35,36,37], we predicted that the dyads engaged in synchronous movement would exhibit greater social bonding with their partner compared to the dyads engaged in asynchronous movement.

Openness, the trait of intellectual curiosity, imagination, and seeking out new experiences and new ideas [38], decreases in older age [39,40,41] and may be reflected by the shrinking social networks of family, friends, neighbors, and community members in old age [42]. Social networks reach a peak at the age of 25 and decrease steadily after the age of 55 [43]. Older persons are less likely to develop new relationships [44]. According to Carstensen’s socioemotional selectivity theory [45], although social networks steadily decline in old age, older adults invest more efforts in close social partners (e.g., family and friends) and prefer to nurture deep, strong, and long-lasing bonds with loved ones in order to maintain their emotional well-being [46,47]. Older adults also demonstrate decreased openness, potentially leading them towards an appreciation for traditional norms [48]. In contrast, young adults are more open to new experiences and diverse perspectives, often forming numerous social connections compared to older adults [38]. Therefore, we assumed that an unfamiliar lab task might be perceived as threatening by older adults, consequently leading them to be less inclined to perceive closeness with others, compared to young adults (regardless of movement synchronicity and in-age-group or out-age-group combination).

A growing body of research explores the intersection between movement synchronicity and group processes. Previous research demonstrated greater cooperation and social connection following synchronous vs. asynchronous movements with outgroup members [31,32,33]. For example, a study’s findings demonstrate that, when two participants from different cultural backgrounds were primed to perceive each other as belonging to an outgroup, they cooperated much better in the game that was played after a synchronous task compared to the game played after an asynchronous task [31]. Moreover, in the absence of intergroup competition, it was found that people exhibited greater spontaneous synchronicity when performing a movement task with members of a different social group compared to members of the same group [33]. Another study examined how synchronized movement affects cooperation between distinct groups, marked by red or blue uniform [32]. The participants wore headphones and were requested to tap to the beat. Six participants were randomly assigned to one of three synchrony conditions: intergroup synchrony (they taped to the same beat), intragroup synchrony (they tapped to a different beat), or asynchrony (no specific tapping pattern). Following this procedure, they played a game and the level of their cooperation in this game was measured. The participants who taped in synchrony with the outgroup demonstrated more cooperation compared to the other two conditions. These findings suggest that moving in synchrony, even with outgroup members, can promote a sense of interpersonal closeness, as demonstrated in the increased cooperation with outgroup members. Based on these studies, we hypothesized that participants who danced in synchrony with a partner from a different age-group would report feeling a greater outgroup overlap (i.e., greater closeness between generations).

Following this literature review, the following research hypotheses were formulated:

**H1.**  *Synchronized dyads will be associated with higher levels of social bonding with the dyadic partner in comparison to asynchronized dyads.*

**H2.**  *Young adults, compared to older adults, will report a greater increase in perceived closeness between generations. That is, following any social encounter (whether with the in-age-group or with the outgroup, whether synchronized or asynchronized).*

**H3.**  *The highest reported closeness between generations is expected to be among participants dancing in synchrony with an out-age-group member. A lower degree of reported closeness between generations is expected among participants dancing in synchrony with an in-age-group member. The lowest level of reported closeness between generations is expected to be among participants dancing asynchronously, with an in-age-group or out-age-group member.*

## 2. Materials and Methods

### 2.1. Sample

A convenience sample, using a snowball sampling method, via social media (e.g., Facebook and WhatsApp), of 168 community-dwelling Jewish Israeli older adults (M = 72.33, age range: 65–90 years) and young adults, i.e., university students (M = 27, age range: 20–45 years) from central and southern Israel, was recruited. While there is some variation in the definition of young adulthood, it generally encompasses individuals between 18 and 45 years of age [49]. Although debated, in Western societies, 65 years is frequently used as the starting point of old age [50]. Therefore, the present study focused on these two age ranges. The inclusion criteria were age, proficiency in Hebrew, and lack of physical and cognitive limitations (the participants reported whether they had been diagnosed with physical or cognitive impairments). Twelve participants who did not meet these criteria were excluded. The final sample included 80 older adults (M = 72.33, SD = 5.83, 72.5% women) and 88 young adults (M = 27, SD = 5.77, 70.5% women). In terms of education, 10.1% had completed elementary school, 31.5% had completed high school, 17.3% had a degree above the high school level, and 41.1% had an academic degree. In terms of marital status, 46.4% were married. Most of them reported good (41.1%) and very good health (47.6%), and the rest reported pretty good health (9.5%) and bad health (1.8%). One third (36.9%) defined their economic status as average, while over half (54.2%) stated that their economic status was above-average and the rest (9.5%) a below-average economic status. In terms of religiosity, the levels were almost evenly distributed between secular, traditional, or religious (33.3%, 29.2%, and 28%, respectively), whereas the rest (9.5%) defined themselves as ultra-orthodox.

After recruitment, the participants were randomly assigned to one of six dyadic conditions, according to age-group affiliation (in-age-group vs. out-age-group) and synchrony (synchronous movements vs. asynchronous movements): Condition 1, young and older adults/synchronous movements (*n* = 26); Condition 2, young and older adults/ asynchronous movements (*n* = 30); Condition 3, young and young adults/synchronous movements (*n* = 34); Condition 4, young and young adults/asynchronous movements (*n* = 32); Condition 5, older and older adults/synchronous movements (*n* = 26); and Condition 6, older and older adults/asynchronous movements (*n* = 20). This research received approval by the Research Ethical Committee at the authors’ university (no. 0221).

### 2.2. Procedure

Data were collected from March 2022 to February 2023 by the first author. Before the experiment, the participants completed a baseline questionnaire delivered electronically via Qualtrics, a secure and user-friendly online survey platform. A week later, two participants, new to each other, met in the lab for an 11 min joint activity. The participants were unaware of their assigned condition (synchronous or asynchronous). First, they watched an instruction video clip in which a dancer sitting on a chair performed four hands movements (adapted from [10]). They were asked to mimic and memorize the sequence of the movements. Afterwards, they were seated on two chairs facing each other one-and-a-half-meters apart. The experimenter provided the following instructions: “Now we will begin an experiment in which you are asked to look at the other participant and move in time with the music and according to the instructions you will hear. Your goal is to stick to the rhythm of the music you will hear. You will not know what the next movement will be, but when you hear the instructions, finish the movement you are making and start the next one while maintaining the rhythm and moving along with the music. If your active hand hurts, you can switch hands to make it easier. Throughout the experiment, we are kindly asking you not to talk with your partner. Please continue to perform movements throughout the task. The joint dance will last 11 min. When we say the request ‘start’, please, start immediately”. During the experiment, the participants listened separately to recorded instructions through individual earphones. The music, composed by the first author, was a syncopated upbeat of percussion and drums. We opted for a sequence of drumbeats (as opposed to familiar songs; [10]) to minimize emotional arousal stemming from past experiences. The participants were instructed to look at their partner and move their hands according to the recorded instructions and the beat. Under the synchronous condition, both participants listened to music at the same pace (110 beats per minute [BPM]), whereas under the asynchronous condition, they listened to the same music but at a different pace (90/110 BPM). The use of a different pace did not allow the movements of the participants to be aligned and thereby created asynchronous movements. The specific tempi were selected following previous studies which found a link between tempo and groove perception, with faster tempos often associated with higher groove ratings [51,52]. Janata et al. [51] found that the average tempo of stimuli rated as having the most groove was 115.6 BPM. Other studies estimated that the tempo for the experience of groove is within a range of 107 to 126 BPM [53,54]. The lower tempo range was chosen to avoid participants’ hands fatigue. Afterward, the participants completed questionnaires and were debriefed. They were thanked and granted USD 15 for their time and effort.

### 2.3. Instruments

#### 2.3.1. Social Bonding

Social bonding was assessed using items adapted from previous research [27,28,29]. The participants rated their level of bonding with their dance partner on a scale ranging from 1 = “not at all” to 7 = “very much”, in response to six questions (i.e., how enjoyable was dancing with the partner? how close did you feel? how much did you like the partner? how similar did you feel to the partner? how much did you trust the partner? how much would like to know the partner?). The items were averaged, with higher scores depicting higher social bonding. The overall reliability of the scale was very good (Cronbach’s alpha = 0.86).

#### 2.3.2. Ingroup–Outgroup Overlap

**Figure 1:**
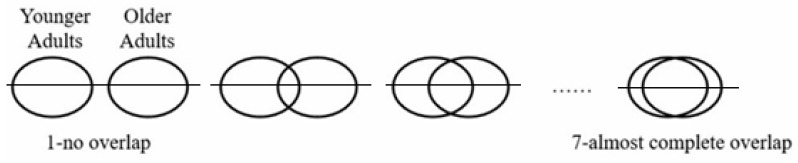
Perceived closeness between generations measured by the levels of overlap between the two social groups.

The ingroup–outgroup overlap [55] scale is based on the Inclusion of the Other in the Self (IOS) [56], which depicts two equal circles with increasing areas of overlap. It was designed as a way to depict perceived closeness between groups and also consider components such as teamwork and time together [57]. These qualities of the IOS made it appropriate for measuring the level of perceived closeness between young and older adults (see also [58]), who participated in a joint effort to synchronize their movements according to the pace of music during an 11 min interaction. The overlapping levels between the two groups, which indicate the closeness level, were coded from 1 (no overlap) to 7 (highest overlap) between the two groups. The participants provided their evaluations twice (a week before and after the intervention). Higher scores depicted greater intergenerational closeness, as shown in Figure 1. They also completed three cover items: the overlap on the same scale of “secular” vs. “religious”, “non-European origin” vs. “European origin” (“Mizrachi” vs. “Ashkenazi” in Israeli terms), and “left” vs. “right” (political stance).

### 2.4. Data Analysis

The statistical analyses were performed by SPSS 28. Preliminary comparisons of the background characteristics were examined. Prior to testing the hypotheses, we examined the differences across the participants by means of age-group affiliation. Specifically, we applied the two-independent sample t-test for continuous or ordinal measurements, and the chi-square test of frequency distribution for the categorical or nominal variables. As expected, we found a higher (better) health condition among the younger adults compared to the older adults (M[mean]_difference_ = 0.28, t(135.87) = 2.15, *p* = 0.013, ES [Cohen’s *d* effect size] = 0.39), a higher economic status among the older adults (M_difference_ = 0.28, t(166) = −7.30, *p* < 0.001, ES = −1.13), and a higher religiosity level (M_difference_ = 0.28, t(142.45) = −2.22, *p* = 0.014, ES = −0.34) among the older adults. We did not find education differences between the older and younger adults. In addition, the mean of the three baseline social closeness items was compared (IOS—social groups), and no difference was found between young and older adults (M_difference_ = 0.28, t(166) = 1.46, *p* = 0.15, ES = 0.19). Moreover, no difference was found when comparing the item measuring the age-group social closeness between young and older adults (M_difference_ = 0.114, t(166) = 0.53, *p* = 0.34, ES = 0.21). These comparisons resulted in no age-group differences on average. In other words, both young and older participants showed similar perceived differences on average; see further explanation of these closeness measures in the next section. Altogether, these preliminary comparison results confirmed the random design of the experiment. We found the women–men ratio to remain similar in both age groups (Pearson’s X(1)^2^ = 0.086, *p* = 0.769), as was the in-relationship versus the not-in-relationship frequency difference (Pearson’s X(1)^2^ = 1.43, *p* = 0.232). These results led to minimizing the further modeling framework to the necessary background variables to control for possible confounding effects. Specifically, we kept health condition, economic status, religiosity, and perceived closeness, beyond synchronization, congruency (i.e., in/out-age-group), and age-group affiliation (i.e., 20–45/65–90) as the controls. Note that all the comparisons were applied on the pre-intervention data. Additionally, the research data did not consider the gender composition of the dyads, as the limited sample size did not allow control over this variable.

To test the three hypotheses, we performed a generalized estimating equation (GEE) modeling approach to integrate the dyadic structure of the experiment. Specifically, the participants operated the various assignments in pairs, thus assigning two sources to the total variance: variation across individual measurements (level one data) and variation across dyads (level two data), where the latter was expressed in the dyadic random intercept (the working correlation matrix; [59]). Wald’s X^2^ test, which assesses the significance of parameters (coefficients) estimated in a statistical model, was used to assess the between-dyad significant differences, e.g., synchronized versus asynchronized dyads, in-age-group versus out-age-group dyads. Predicted marginal means (i.e., average predicted score for a specific variable) were calculated and compared. To complete the model, personal characteristics were added to the model, e.g., health condition, economic status, religiosity, and perceived closeness.

## 3. Results

To test the first hypothesis, a GEE model was constructed, with social bonding as the post-intervention outcome. The explanatory set included the dyadic synchronous versus asynchronous dyads and the in-age-group versus out-age-group dyads. Other individual-level indicators were age-group and health condition, economic status, and religiosity. The synchronization condition was found to affect social bonding (b = 0.51, *p* = 0.014), such that the synchronized cases were associated with higher levels of social bonding with the dyadic partner in comparison to the asynchronized cases (predicted means: M_synchrony_ = 3.52, 95%CI [3.19, 3.85]; M_asynchrony_ = 3.01, 95%CI = [2.78, 3.25]). No other independent effects were found to be significant. These model results lend support to the first hypothesis (see Table 1).

A similar GEE model was built to analyze the change in intergeneration closeness from pre to post intervention. Table 2 provides the GEE model results for the H2 test. We looked at the age-group effect on the change in perceived age closeness, controlled by the perceived closeness pre intervention. The results lend clear support to H2: that is, younger adults experienced a greater and positive change in their age closeness perception in comparison to older adults (b = 0.69, *p* < 0.001; M_younger_ = 0.62, SE = 0.13, 95%CI = [0.36, 0.88]; M_older_ = −0.07, SE = 0.16, 95%CI = [−0.37, 0.24]). In addition, the pre-intervention closeness was negatively associated with the change, regardless of the participants’ age (b = −0.56, *p* < 0.001), representing a regression to the mean, as explained above.

**Figure 2:**
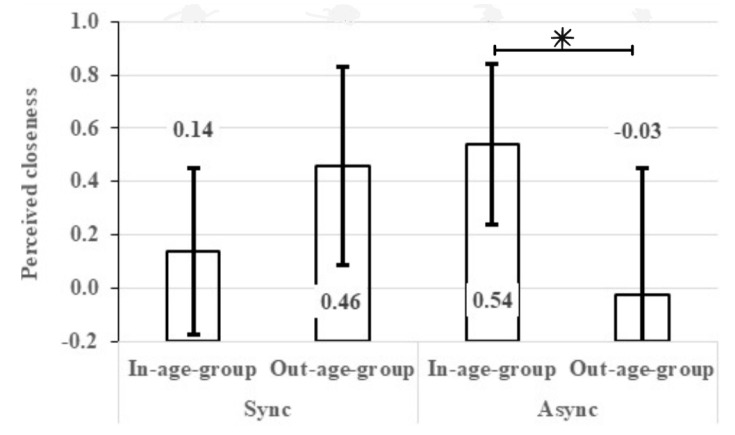
Change in perceived closeness between generations: interaction of synchronization condition and in/out-age-group. Note: The asterisk indicates the significant difference (*p* = 0.020) between the in-age-group and the out-age-group within the asynchronous condition based on multiple comparisons with a Bonferroni correction. The participants who moved out of synchrony with members of a different age-group showed a negative perceived closeness between generations, compared with the participants who moved out of synchrony with their in-age-group members.

To test the moderation hypothesis (H3), we tested a series of two-way interactions as well as a composite three-way interaction. These interaction effects were tested beyond the main effects of the above factors and the other covariates included in the previous model. Table 3 shows that synchronization interacted with age-congruency to affect the change in perceived closeness (Wald = 5.40, *p* = 0.020). This interaction is presented in Figure 2. Each bar represents the marginal mean of a subgroup. As predicted and as depicted in Figure 2, the lowest level of change in perceived closeness between generations was found among the participants in the asynchronized out-age-group condition (M_incongruency_ = −0.03, 95%CI= [−0.50, 0.45]). However, both the in-age-group vs. out-age-group marginal means within the synchronized category did not differ from the lowest subgroup marginal mean and the marginal mean of the asynchronized in-age-group, on average. The main difference was found between the in-age-group and out-age-group marginal means within the asynchronized category, on average (M_congruency_ = 0.54, 95%CI = [0.23, 0.84]). More specifically, a negative or an overall mean reduction in perceived closeness between generations was found among the out-age-group asynchronized subgroup, which significantly differed only from the in-age-group within the subgroup category, while the mean value of perceived closeness was positive for the rest of the research respondents. H3 was supported by the interaction, but the findings were contradictory to H3, as the highest reported closeness between generations was found among the participants dancing in asynchrony with an in-age-group member.

## 4. Discussion

This study is the first to examine a way to increase intergenerational social bonding and intergenerational closeness through dance movements. The intervention consisted of a short movement activity (11 min), in synchrony or out of synchrony, between two partners who had no prior acquaintance, with 84 dyads (old with young, young with young, or old with old). This study examined the effect of synchronicity, age-group, and the age structure of a dyad (in- or out-age-group) on the social bonding toward a partner (interpersonal level) and the degree of perceived closeness between generations (social level). Synchronous dance movements resulted in greater social bonding with one’s partner. Young adults (aged 20–45) reported a greater increase in closeness between generations compared to older adults (aged 65–90). Unexpectedly, an increase in closeness between generations was reported among the participants who danced out of sync with their in-age-group compared to those who danced out of sync with the out-age-group.

The first finding of this study resonates with numerous research studies establishing the social benefits arising from the performance of synchronized movements [10,18,23,25,26,27,28,29,30,31,32,33,34,35,36,37]. Synchronized movements have been shown to create a sense of camaraderie within both ingroups and outgroups [30,31,32,33], termed “social glue”. Synchrony has been shown to promote social bonds among toddlers [60], children [14,61], adolescents [62], adults [10], and even toward virtual avatars [63]. Yet, our study is the first to explore the influence of movement synchronization on social bonding in an intergenerational context. The participants allocated in the in-age-group dyads (young–young, old–old) and out-age-group dyads (young–old) reported greater social bonding with their dancing partners when the dyadic movements were synchronized. It is important to note that the score of social bonding was based on the mean of items for each dyad. In line with the “chameleon effect” [20] and with the role of rhythm in enhancing coordinated movements [22], in the present study, synchronized partners, entertaining the same musical beat, were more aligned with each other. The same beat enabled them to react swiftly to changes in their partner’s movements by activating their natural tendency to mimic their partners’ movements in order to better communicate with them. Indeed, dyads who performed synchronized as opposed to asynchronized dance movements reported greater social bonding because they enjoyed, felt closer to, found similarity in, liked, trusted, and were keen to know their partner. Such an interpretation can also be supported by another study, which found that a “mirror game”, in which older adult dyads mimicked each other’s movements, increased the level of attention, enjoyment, and responsiveness to the partner [64]. Moreover, this effect of synchronous movements on social bonding was so robust that it included both in-age-group and out-age-group dyads. In other words, simple synchronous movements while sitting for 11 min strengthened interpersonal bonding within and across age groups.

Such processes, which include perception–action coupling [11], are described as breaking down the “boundaries” of the self and enhancing the sense of overlap between the self and the partner, so that the perception of similarity between the two increases [16,17,18]. In this context, it can be reiterated that walking synchronously with a member of an outgroup (belonging to a socially marginalized group) led to a decrease in prejudice towards the outgroup, compared to walking asynchronously [34]. The strong impact of synchronized movement on the individual suggests that future studies should examine whether synchronized movements can also reduce age stereotypes and decrease ageism.

Regarding the effect of asynchronized movement on social bonding, it is possible that the “boundaries” of the self and the partner among the dyads who moved out of synchrony became thicker. When the participants noticed that, despite their efforts, their movements were still not coordinated, they gave up, stopped investing their attention in mimicking their partner, and started to focus on their own movements. Thus, the non-synchronized participants demonstrated less social bonding to the partner. Later in this paper, upon discussing hypothesis 3, we will elaborate more about the psychological experience of these participants.

Our findings also confirm the second research hypothesis regarding the propensity of young adults to increase perceived closeness between generations, which was not expected from the older adults. Following the intervention, the young adults probably exhibited positive “social flexibility” regardless of the synchrony condition or the age composition of their dyad and reported feeling greater closeness between young and older adults. This finding is also consistent with a systematic review that examined the effectiveness of 63 intergenerational intervention programs with over 6100 participants, finding them more effective for young adults [7]. Why, then, did young people demonstrate greater social flexibility? It should be noted that the average age of the young adults who participated in the experiment was 27 years, and most of them were university students, whose lifestyle invites exposure to many people and new and diverse ideas. The lack of change in intergenerational closeness among the older adults may also be attributed to the decline in social networks [44] and decreased openness that develop with advanced age [38]. While young adults’ socialization is future-oriented, and they tend to exhibit greater openness to novel social encounters [43], time horizons shrink in old age, and older adults prioritize cultivating deeper, long-lasting relationships with established social networks while limiting their exposure to less meaningful new ties [45,46,48,65]. The current study employed a relatively brief, 11 min intergenerational dance activity, which did not consider these developmental differences. This duration could have been too short for observing significant increases in closeness among older adults, as the formation of a deeper bond may require more sustained interaction and continuous engagement.

The intervention’s effectiveness could also be modulated by cohort effects and age stereotypes. Cohorts are defined as groups of individuals born around the same time and sharing similar experiences [66]. According to the literature, older adults who are currently in their 70s and 80s might hold more conservative values due to their historical context [66]. Current younger generations grew up with developed social networks and might, therefore, be more open to new social encounters, such as dancing which fosters intergenerational connections. Moreover, both young and older people may hold stereotypical views of one another (e.g., millennials/Gen Z perceived as less respectful, older persons perceived as less tech-savvy) [67]. These cohort differences and age stereotypes can influence the willingness of individuals to interact with an out-age-group member. The lack of measurement of such pre-existing age stereotypes is a limitation of the current study. Future studies can incorporate measures of such age stereotypes and explore how these beliefs might moderate the perceived closeness between generations.

The significant interaction effect of age-group X synchronicity on intergenerational closeness was opposite to hypothesis 3. It indicated that participants who danced with their in-age-group partner asynchronously reported increased closeness between generations (see Figure 2) compared to those who danced with their out-age-group partner asynchronously. Ostensibly, asynchronous movement may cause increased social distancing, since it demonstrates unsuccessful social interaction, which creates a kind of “psychological buffer” between the participants. Yet, intergroup dynamic theories may provide an explanation for the findings. Social identity theory [68] posits that individuals seek positive distinctiveness for their ingroups by comparing them favorably to other groups, a phenomenon termed as “ingroup favoritism”. Individuals tend to favor their ingroup due to the motivation to maintain positive self-esteem in an intergroup context (“we are better than them”). In the context of the present study, it is possible that the asynchronous movements with an in-age-group partner (young with young and old with old) reduced the favoritism for the in-age-group because the dance was frustrating. When the participants noticed that despite their efforts, their movements were still not aligned with members of their in-age-group, the effect of perceived similarity to people of their age-group diminished, which led to a mitigation of the ingroup favoritism bias. Thus, the decrease in the ingroup favoritism bias maintained the positive value of the out-age-group, which was reflected in the reporting of increased closeness between generations.

As expected, asynchronous movements with the outgroup increased the perceived distance between generations (the negative value in right column; Figure 2). It is possible that movement limitations that were apparent among older adults, who, according to the instructions, tried in vain, in the asynchronous situation, to synchronize with their young partner, led the young participants to focus on negative aging cues. Due to their failure to achieve synchronous movements in the asynchronous condition, older persons could be portrayed by young adults as clumsy and disabled. The older people in these asynchronous dyads may have sensed the discomfort that their young partners could experience due to the asynchrony of the dance movements and felt frustrated because they had troubles adjusting to their young partners and could attribute these difficulties to their advanced age. In sum, it is possible that intergenerational dancing with the outgroup that was not synchronized led all participants to perceive intergenerational distance, whereas asynchronized intergenerational dancing with the in-age-group could mitigate ingroup favoritism.

Future replications of this design can incorporate additional measures, particularly those related to awareness of age-related changes. This concept measures the experiences that make people aware that their behavior, level of performance, or ways of experiencing their lives have changed with aging [69] and is an important concept for understanding views of aging [70]. During the direct face-to-face dance, older adults’ movements could have been noticed by their partners as cues of aging (e.g., less flexibility and more vulnerability of the aging body) and have activated the former’s awareness of age-related changes. Such awareness could have been painful for them and, therefore, decreased their willingness for intergenerational closeness. Such effects could have been more evident among older adults with more awareness of the aging period as a period of losses and who might be more sensitive to the motoric differences between generations. Therefore, future studies may want to either control this variable or use it as a moderator factor in the relationship between synchronous movement and intergenerational closeness. Additionally, to validate the argument that asynchronized intergenerational dancing with the in-age-group can mitigate ingroup favoritism and, thereby, create intergenerational closeness, a measure of in-age-group closeness (e.g., in-generational closeness measured by the inclusion of the ingroup in the self [71]) can be used. Finally, in order to examine the effect of greater intergenerational closeness among younger adults, future studies can also add a measure of openness to experience, a personality trait associated with willingness to engage with novel experiences.

The findings of the current study should be discussed while considering its limitations. First, since it was carried out in the lab, it lacks external validity. Data collection occurred primarily in a university laboratory, which could have been more familiar to the young participants (who were students) than the older participants. This context could decrease the comfort level of older adults and, consequently, their judgments regarding their perceived closeness to young adults as a group. This problem of external validity can be balanced if the experiment were to be conducted in daycare centers, or even in ceremonial contexts (e.g., weddings or festivals), using changes in tempo or dynamics which may produce creative physical interactions. Second, future studies need to recruit larger, homogeneous, and random samples. In this regard, the relatively small number of participants in each of the six experimental conditions and the high percentage of women did not allow us to examine the effect of the dyad structure in terms of gender. However, this problem also appears in other studies (see [10,27,28]). Third, due to the complexity of the study design, similar to other studies, this study did not measure the effect of alternative activities within dyads (e.g., conversations, working together on a shared cooperative task; see [10,27,28]). Fourth, this study did not examine the stability of the effects over time (e.g., [72]). Finally, measuring physiological measures that signify synchronization (e.g., inter-beat intervals of heartbeats to test the synchronization between participants, see [73]) or closeness (e.g., saliva samples to measure oxytocin secretion) can add understanding to the mediating physiological mechanisms of synchronization, leading to intergenerational closeness.

## 5. Conclusions

This study offered a novel perspective on group dynamics, as it demonstrated that intergenerational activities of synchronized movements, such as dancing, may increase social cohesion and strengthen interpersonal relationships. Practitioners from various fields—welfare and music—may implement intergenerational rhythmic activity. Of particular importance is the finding that asynchronous movement within age groups fostered intergenerational closeness, as it might indicate that asynchronized in-age-group interaction might weaken favoritism towards one’s own age-group and encourage closeness to the out-age-group.

Our findings also suggest that future intergenerational interventions may focus on people of younger age populations. In terms of perceived intergenerational closeness, intergenerational interventions may be more beneficial for young people. This conclusion is derived from younger participants in this study reporting that they felt closer to older adults (a main effect which was not affected by synchrony or the in-age-group or out-age-group dyads). Moreover, exploring the effectiveness of such interventions among younger people across diverse social groups could be a valuable avenue for future research.

Our findings emphasize the importance of integrating such activities into intergenerational programs while testing their effectiveness using additional psychological measures, including ingroup favoritism, openness to experience, and different cohort effects (e.g., baby boomers vs. Gen Z). Additionally, future studies should examine *how* such activities bridge generations. Therefore, it is important to explore several mediators that may serve as potential mechanisms for intergenerational closeness, such as subjective age, i.e., the age one feels [74], and physical self-efficacy [75]. A successful synchronization with a younger partner might decrease older adults’ subjective age and, thereby, increase the perceived closeness between generations following participation in a joint activity. In a similar vein, it can temporarily improve older adults’ physical self-efficacy, increasing the perceived closeness between generations. Future studies that explore these avenues could provide valuable insights with practical implications for fostering intergenerational connections that bridge the gap created by age segregation. Therefore, this interdisciplinary study opens the door to future research and presents a promising first step on the way to developing programs that will challenge age-related biases by bringing generations closer together.

## Figures and Tables

**Table 1:** GEE modeling results for social bonding by synchronization and congruency.

	B	SE(b)	95%CI	Wald’s Chi-Square	Sig.
Synchrony (sync vs. async)	0.51	0.21	[0.10, 0.91]	6.09	0.014
Congruency (in-age-group vs. out-age-group)	0.09	0.21	[−0.33, 0.50]	0.17	0.683
Age-group (20–45 vs. 65–90)	−0.35	0.23	[−0.80, 0.09]	2.45	0.117
Health condition	0.04	0.14	[−0.25, 0.32]	0.06	0.805
Economic situation	−0.02	0.13	[−0.28, 0.23]	0.03	0.867
Religiosity	0.15	0.09	[−0.03, 0.33]	2.63	0.105
QICC (Quasi-likelihood under independence model criterion)	257.33				

Notes: In square brackets, 95% confidence interval around the point estimate. Predicted marginal means: M_sync_= 3.52, SE = 0.17, 95%CI= [3.19, 3.85]; M_async_ = 3.01, SE = 0.12, 95%CI = [2.78, 3.25].

**Table 2:** GEE model results for perceived age-group closeness by age-group affiliation.

	B	SE (b)	95%CI	Wald’s Chi-Square	Sig.
Synchrony (sync vs. async)	−0.06	0.19	[−0.42, 0.31]	0.10	0.754
Congruency (in-age-group vs. out-age-group)	0.10	0.19	[−0.27, 0.48]	0.30	0.586
Age-group (20–45 vs. 65–90)	0.69	0.21	[0.27, 1.11]	10.39	0.001
Health condition	−0.17	0.16	[−0.49, 0.15]	1.08	0.299
Economic situation	0.14	0.12	[−0.09, 0.37]	1.42	0.234
Religiosity	0.14	0.10	[−0.05, 0.34]	2.10	0.148
Perceived closeness—pre	−0.56	0.07	[−0.70, −0.42]	61.22	<0.001
QICC	275.10				

Notes: In square brackets, 95% confidence interval around the point estimate. Predicted marginal means: M_younger_ = 0.62, SE = 0.13, 95%CI = [0.36, 0.88]; M_older_ = −0.07, SE = 0.16, 95%CI = [−0.37, 0.24].

**Table 3:** Two- and three-way interaction analyses by age-group affiliation, congruency, and synchronization on change in perceived closeness.

	Wald’s Chi-Square	Sig.
Two-way interactions		
Synchrony × Congruency	5.40	0.020
Synchrony × Age-group	0.42	0.517
Congruency × Age-group	0.01	0.914
Three-way interaction		
Synchrony × Congruency × Age-group	0.01	0.913
QICC two-way interaction model	272.73	
QICC three-way interaction model	278.88	

## Data Availability

The data presented in this study are available upon request from the corresponding author.
